# *catena*-Poly[[[diaqua­copper(II)]-bis­[μ_2_-1,3-bis­(1,2,4-triazol-1-yl)propane]] dinitrate monohydrate]

**DOI:** 10.1107/S1600536809035958

**Published:** 2009-09-12

**Authors:** Wen Feng, Xiao-Jun Zhao, En-Cui Yang

**Affiliations:** a College of Chemistry and Life Science, Tianjin Key Laboratory of Structure and Performance for Functional Molecule, Tianjin Normal University, Tianjin 300387, People’s Republic of China

## Abstract

The title Cu^II^ coordination polymer, {[Cu(C_7_H_10_N_6_)_2_(H_2_O)_2_](NO_3_)_2_·H_2_O}_*n*_, was obtained by the reaction of equimolar Cu(NO_3_)_2_·4H_2_O and 1,3-bis­(1,2,4-triazol-1-yl)propane (btp) in a water–methanol solvent. The Cu^II^ atom is located on a centre of inversion and has an elongated octa­hedral coordination geometry formed by four N atoms from four symmetry-related btp ligands and two coordinated water mol­ecules. Adjacent Cu^II^ atoms are connected by btp ligands, generating a double-stranded chain. The nitrate anion is disordered over two positions in a 0.828 (7):0.172 (2) ratio.

## Related literature

For the structures and applications of functional metal complexes in coordination and materials science, see: Blake *et al.* (1999 ▶); Evans & Lin (2001 ▶); James (2003 ▶); Janiak (2003 ▶); Mitzi*et al.* (2001 ▶); Moulton & Zaworotko (2001 ▶); Papaefstathiou & MacGillivray (2003 ▶). For the structures of btp-based metal complexes, see: Wang *et al.* (2006 ▶); Yin *et al.* (2006 ▶); Zhu *et al.* (2009 ▶); Van Albada *et al.* (2000 ▶); Tian *et al.* (2008 ▶); Zhao *et al.* (2002 ▶); Gu *et al.* (2008 ▶).
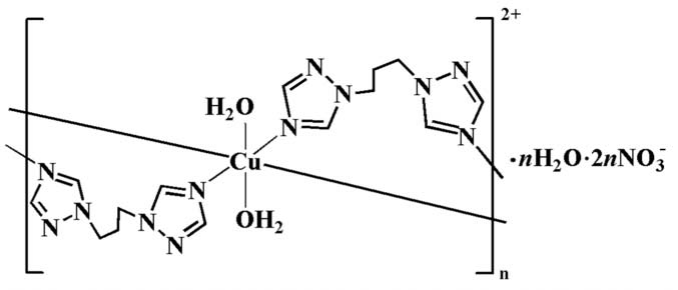


## Experimental

### 

#### Crystal data

[Cu(C_7_H_10_N_6_)_2_(H_2_O)_2_](NO_3_)_2_·H_2_O
*M* *_r_* = 598.03
Monoclinic, 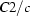

*a* = 11.177 (3) Å
*b* = 12.449 (3) Å
*c* = 17.312 (4) Å
β = 91.655 (4)°
*V* = 2408.0 (9) Å^3^
*Z* = 4
Mo *K*α radiation
μ = 0.98 mm^−1^
*T* = 296 K
0.24 × 0.18 × 0.16 mm

#### Data collection

Bruker APEXII area-detector diffractometer
Absorption correction: multi-scan (*SADABS*; Sheldrick, 1996 ▶) *T*_min_ = 0.798, *T*_max_ = 0.858
5954 measured reflections
2115 independent reflections
1874 reflections with *I* > 2σ(*I*)
*R*_int_ = 0.014

#### Refinement

*R*[*F* ^2^ > 2σ(*F* ^2^)] = 0.041
*wR*(*F* ^2^) = 0.112
*S* = 1.07
2115 reflections
184 parameters
26 restraints
H-atom parameters constrained
Δρ_max_ = 0.93 e Å^−3^
Δρ_min_ = −0.56 e Å^−3^

### 

Data collection: *APEX2* (Bruker, 2003 ▶); cell refinement: *SAINT* (Bruker, 2001 ▶); data reduction: *SAINT*; program(s) used to solve structure: *SHELXS97* (Sheldrick, 2008 ▶); program(s) used to refine structure: *SHELXL97* (Sheldrick, 2008 ▶); molecular graphics: *SHELXTL* (Sheldrick, 2008 ▶) and *DIAMOND* (Brandenburg & Berndt, 1999 ▶); software used to prepare material for publication: *SHELXTL*.

## Supplementary Material

Crystal structure: contains datablocks I, global. DOI: 10.1107/S1600536809035958/bt5049sup1.cif

Structure factors: contains datablocks I. DOI: 10.1107/S1600536809035958/bt5049Isup2.hkl

Additional supplementary materials:  crystallographic information; 3D view; checkCIF report

## Figures and Tables

**Table 1:** Selected geometric parameters (Å, °)

Cu1—N6^i^	1.998 (2)
Cu1—N3	2.035 (3)
Cu1—O4	2.456 (3)

Symmetry code: (i) 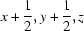.

**Fig. 1.**
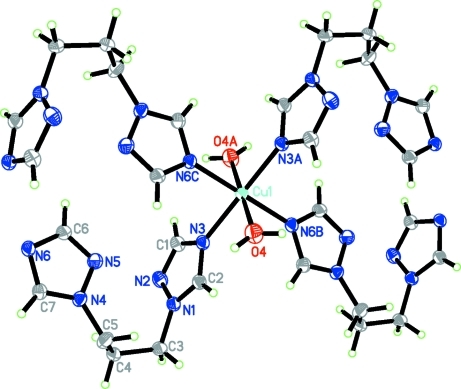
The local coordination environment of the CuII atom in I. Displacement ellipsoids were drawn at 30% probability. [Symmetry codes: (A) –x + 3/2, –y + 1/2, –z, (B) x + 1/2, y + 1/2, z, (C) –x + 2, –y + 1, –z.]

**Fig. 2.**
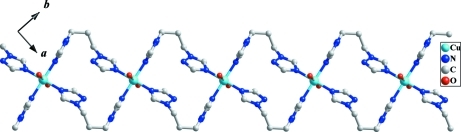
Part of the chain of the title compound.

## supplementary crystallographic information

### Comment

Recently, rapid progress has been made on the the construction and applications of the functional metal complexes in diverse science fields (Blake *et al.*, 1999; Evans *et al.*, 2001; James *et al.*, 2003; Janiak *et al.*, 2003; Mitzi *et al.*, 2001; Moulton *et al.*, 2001; Papaefstathiou *et al.*, 2003). In this regard, 1,3-bis(1,2,4-triazol-1-yl)propane(btp), one of the most popular derivatives of 1,2,4-triazole, has received more and more attention in the fields of coordination chemistry and material science due to its multiple binding sites, flexible skeleton and intense fluorescence emission behavior (Wang *et al.*, 2006; Yin *et al.*, 2006; Zhu *et al.*, 2009). Indeed, bearing two triazolyl groups being connected by a flexible propane linker, btp ligand in the transitional metal complexes has exhibited variable bi- (Van Albada *et al.*, 2000), tri- and tetra-dentate (Tian *et al.*, 2008) coordination modes as well as commonly observed *anti-anti*, *anti*-*gauche*, and *gauche*-*gauche* conformations. Thus, a variety of interesting structures ranged from the discrete binuclear, infinite one-dimensional *Z*-shaped, ladder-like, double-, and triple-stranded chains to two-dimensional grid-like layer, have been generated. Obviously, the structural diversity of the btp-based metal complexes depends strongly on the binding features of the metal ions and the functional ligands. Herein, to further investigate the binding behavior of the btp ligand, a double-stranded Cu^II ^coordination chain, (I), was obtained by the reaction of Cu(NO_3_)_2_. 4H_2_O and btp in mixed water-methanol medium.

X-ray structural analysis reveals that I consists of a one-dimensional double-stranded cationic chain and a disorder NO_3_^- ^for charge compensation. The Cu^II^ atom locates at special position and is in an elongated octahedral coordination geometry constructed by four triazole nitrogen atoms (N3, N3A, N6B, N6C) from four symmetry-related btp ligands in an equatorial plane and two coordinated water molecules occupying the apical positions (see Figure 1). The Cu–N distances are *ca*. 0.5 Å shorter than that of Cu–O separation due to the Jahn–Teller effect (see Table 1).

Pairs of neutral btp ligands adopt an *exo*-bidentate (*µ*_2_-btp-*κ*^1^N^4^:*κ*^1^N^4'^) binding mode to connect the adjacent Cu^II^ atoms into an infinite double-stranded chain along the diagonal of the crystallographic *ab* plane (see Figure 2). As a result, the closed 20–membered [Cu_2_(btp)_2_] metallomacrocycles are alternately generated with the nearest Cu···Cu separation of 8.3654 (13) Å. Such the polymeric chain has ever been obtained in the complexes of [Co(btp)_2_(NCS)_2_]_n_ (Zhao *et al.*, 2002), [Fe(btp)_2_(NCS)_2_]_n_ (Gu *et al.*, 2008), and [Zn(btp)_2_(dca)_2_]_n_ (Zhu *et al.*, 2009), although the metal center and the coligand are different from each other. Notably, the torsion angles of N1/C3/C4/C5 and C3/C4/C5/N4 are -65.695 (18)° and 108.753 (16)°, respectively. And the dihedral angle between the two triazole rings is 84.057 (16)°, which suggests a scarcely observed *gauche-eclipsed *conformation of the btp ligand.

### Experimental

To an aqueous solution (10 ml) of Cu(NO_3_)_2_^.^4H_2_O (30.8 mg, 0.1 mmol) was slowly added a methanol solution (10 ml) of btp (17.8 mg, 0.1 mmol) with constant stirring. The resulting mixture was further stirred for half an hour and then filtered. Upon slow evaporation of the filtrate at room tempreture, blue block-shaped crystals suitable for single-crystal X-ray diffraction analysis were isolated directly within two weeks, washed with ethanol and dried in air (yield: 40% based on Cu^II ^salt). Elemental analysis calculated for C_7_H_13_Cu_0.5_N_7_O_4.5_: C, 28.12; H, 4.38; N, 32.79%; found: C, 28.22; H, 4.29; N, 32.61%.

### Refinement

H atoms were included in calculated positions and treated as riding atoms, with C–H = 0.93 (aromatic) or 0.97 (methylene) Å and O–H = 0.85 Å. All H atoms were allocated displacement parameters related to those of their parent atoms [*U*_iso_(H)= 1.2*U*_iso_ (C), *U*_iso_(H)= 1.5*U*_iso_ (O)]. The nitrate disordered over two positions with a site occupation factor  of 0.828 (7) for the major occupied site. The position of the highest peak is at (0.2337, 0.4251, 0.2774), 1.06Å from O3, and the position of the deepest hole is at (0.1726, 0.3718, 0.2796), 0.39 Å from O3'.

### Crystal data

**Table:**

[Cu(C_7_H_10_N_6_)_2_(H_2_O)_2_](NO_3_)_2_·H_2_O	*F*(000) = 1236
*M**_r_* = 598.03	*D*_x_ = 1.650 Mg m^−^^3^
Monoclinic, *C*2/*c*	Mo *K*α radiation, λ = 0.71073 Å
*a* = 11.177 (3) Å	Cell parameters from 3805 reflections
*b* = 12.449 (3) Å	θ = 2.5–27.7°
*c* = 17.312 (4) Å	µ = 0.98 mm^−^^1^
β = 91.655 (4)°	*T* = 296 K
*V* = 2408.0 (9) Å^3^	Block, blue
*Z* = 4	0.24 × 0.18 × 0.16 mm

### Data collection

**Table:**

Bruker APEXII area-detector diffractometer	2115 independent reflections
Radiation source: fine-focus sealed tube	1874 reflections with *I* > 2σ(*I*)
graphite	*R*_int_ = 0.014
phi and ω scans	θ_max_ = 25.0°, θ_min_ = 2.4°
Absorption correction: multi-scan (*SADABS*; Sheldrick, 1996)	*h* = −13→13
*T*_min_ = 0.798, *T*_max_ = 0.858	*k* = −14→14
5954 measured reflections	*l* = −20→14

### Refinement

**Table:**

Refinement on *F*^2^	Primary atom site location: structure-invariant direct methods
Least-squares matrix: full	Secondary atom site location: difference Fourier map
*R*[*F*^2^ > 2σ(*F*^2^)] = 0.041	Hydrogen site location: inferred from neighbouring sites
*wR*(*F*^2^) = 0.112	H-atom parameters constrained
*S* = 1.07	*w* = 1/[σ^2^(*F*_o_^2^) + (0.0519*P*)^2^ + 7.175*P*] where *P* = (*F*_o_^2^ + 2*F*_c_^2^)/3
2115 reflections	(Δ/σ)_max_ < 0.001
184 parameters	Δρ_max_ = 0.93 e Å^−^^3^
26 restraints	Δρ_min_ = −0.56 e Å^−^^3^

### Special details

**Table:**

Geometry. All e.s.d.'s (except the e.s.d. in the dihedral angle between two l.s. planes) are estimated using the full covariance matrix. The cell e.s.d.'s are taken into account individually in the estimation of e.s.d.'s in distances, angles and torsion angles; correlations between e.s.d.'s in cell parameters are only used when they are defined by crystal symmetry. An approximate (isotropic) treatment of cell e.s.d.'s is used for estimating e.s.d.'s involving l.s. planes.
Refinement. Refinement of *F*^2^ against ALL reflections. The weighted *R*-factor *wR* and goodness of fit *S* are based on *F*^2^, conventional *R*-factors *R* are based on *F*, with *F* set to zero for negative *F*^2^. The threshold expression of *F*^2^ > σ(*F*^2^) is used only for calculating *R*-factors(gt) *etc*. and is not relevant to the choice of reflections for refinement. *R*-factors based on *F*^2^ are statistically about twice as large as those based on *F*, and *R*- factors based on ALL data will be even larger.

### Fractional atomic coordinates and isotropic or equivalent isotropic displacement parameters (Å^2^)

**Table:**

	*x*	*y*	*z*	*U*_iso_*/*U*_eq_	Occ. (<1)
Cu1	1.0000	0.5000	0.0000	0.0316 (2)	
N1	1.1092 (2)	0.1933 (2)	0.06359 (16)	0.0348 (6)	
N2	1.1152 (3)	0.1788 (2)	−0.01363 (17)	0.0422 (7)	
N3	1.0493 (2)	0.3437 (2)	0.01382 (15)	0.0337 (6)	
N4	0.8317 (2)	0.0666 (2)	0.09733 (15)	0.0341 (6)	
N5	0.8401 (2)	0.1305 (2)	0.03421 (16)	0.0415 (7)	
N6	0.6637 (2)	0.0474 (2)	0.03457 (16)	0.0339 (6)	
C1	1.0779 (3)	0.2711 (3)	−0.0409 (2)	0.0407 (8)	
H1	1.0716	0.2857	−0.0936	0.049*	
C2	1.0699 (3)	0.2906 (2)	0.07910 (19)	0.0350 (7)	
H2	1.0585	0.3178	0.1284	0.042*	
C3	1.1409 (3)	0.1067 (3)	0.1175 (2)	0.0405 (8)	
H3A	1.1547	0.1370	0.1686	0.049*	
H3B	1.2151	0.0740	0.1018	0.049*	
C4	1.0458 (3)	0.0208 (3)	0.1216 (2)	0.0386 (8)	
H4A	1.0769	−0.0380	0.1531	0.046*	
H4B	1.0297	−0.0070	0.0700	0.046*	
C5	0.9283 (3)	0.0588 (3)	0.1550 (2)	0.0433 (8)	
H5A	0.9050	0.0093	0.1951	0.052*	
H5B	0.9406	0.1287	0.1787	0.052*	
C6	0.7370 (3)	0.1162 (3)	−0.0014 (2)	0.0382 (7)	
H6	0.7158	0.1505	−0.0476	0.046*	
C7	0.7269 (3)	0.0179 (3)	0.0965 (2)	0.0376 (7)	
H7	0.7015	−0.0299	0.1339	0.045*	
N7	0.2452 (3)	0.3129 (3)	0.2674 (2)	0.0646 (10)	
O1	0.293 (2)	0.2228 (13)	0.2776 (16)	0.147 (3)	0.172 (7)
O2	0.1369 (11)	0.3192 (19)	0.2856 (14)	0.1018 (18)	0.172 (7)
O3	0.2982 (18)	0.3883 (13)	0.2421 (14)	0.229 (6)	0.172 (7)
O1'	0.2728 (6)	0.2639 (6)	0.3235 (3)	0.147 (3)	0.828 (7)
O2'	0.3137 (4)	0.3107 (5)	0.2130 (3)	0.1018 (18)	0.828 (7)
O3'	0.1580 (7)	0.3633 (8)	0.2599 (3)	0.229 (6)	0.828 (7)
O4	0.9447 (3)	0.5006 (2)	0.13626 (15)	0.0523 (7)	
H4'	0.9082	0.4529	0.1616	0.078*	
H4"	0.9664	0.5569	0.1606	0.078*	
O5	0.0000	0.6545 (3)	0.2500	0.0667 (11)	
H5	0.0564	0.7003	0.2495	0.100*	

### Atomic displacement parameters (Å^2^)

**Table:**

	*U*^11^	*U*^22^	*U*^33^	*U*^12^	*U*^13^	*U*^23^
Cu1	0.0248 (3)	0.0273 (3)	0.0426 (3)	−0.0009 (2)	−0.0023 (2)	0.0017 (2)
N1	0.0295 (13)	0.0296 (14)	0.0451 (16)	0.0009 (11)	−0.0017 (11)	0.0012 (12)
N2	0.0463 (16)	0.0349 (15)	0.0457 (17)	0.0025 (13)	0.0070 (13)	−0.0027 (13)
N3	0.0293 (13)	0.0293 (13)	0.0426 (15)	−0.0003 (11)	−0.0003 (11)	0.0004 (12)
N4	0.0284 (13)	0.0361 (14)	0.0377 (14)	−0.0019 (11)	−0.0006 (11)	−0.0006 (12)
N5	0.0334 (14)	0.0441 (16)	0.0468 (16)	−0.0076 (13)	−0.0021 (12)	0.0088 (13)
N6	0.0278 (13)	0.0299 (14)	0.0438 (15)	−0.0016 (11)	−0.0010 (11)	0.0004 (12)
C1	0.0440 (19)	0.0373 (18)	0.0409 (18)	−0.0026 (15)	0.0050 (15)	0.0000 (15)
C2	0.0314 (16)	0.0310 (16)	0.0424 (18)	0.0002 (13)	−0.0004 (13)	−0.0018 (14)
C3	0.0303 (16)	0.0353 (17)	0.055 (2)	0.0033 (14)	−0.0073 (14)	0.0066 (16)
C4	0.0363 (17)	0.0300 (16)	0.049 (2)	0.0015 (14)	−0.0071 (15)	0.0054 (14)
C5	0.0358 (18)	0.055 (2)	0.0386 (18)	−0.0058 (16)	−0.0068 (14)	0.0005 (16)
C6	0.0337 (17)	0.0366 (17)	0.0443 (18)	−0.0014 (14)	−0.0027 (14)	0.0063 (15)
C7	0.0327 (17)	0.0372 (18)	0.0428 (18)	−0.0054 (14)	0.0007 (14)	0.0049 (14)
N7	0.075 (3)	0.070 (2)	0.049 (2)	0.006 (2)	−0.0036 (18)	−0.0077 (19)
O1	0.186 (6)	0.172 (6)	0.086 (4)	0.005 (5)	0.028 (4)	0.069 (5)
O2	0.113 (4)	0.117 (4)	0.077 (3)	0.031 (3)	0.038 (3)	0.014 (3)
O3	0.254 (9)	0.362 (13)	0.073 (4)	0.241 (10)	0.020 (5)	−0.005 (6)
O1'	0.186 (6)	0.172 (6)	0.086 (4)	0.005 (5)	0.028 (4)	0.069 (5)
O2'	0.113 (4)	0.117 (4)	0.077 (3)	0.031 (3)	0.038 (3)	0.014 (3)
O3'	0.254 (9)	0.362 (13)	0.073 (4)	0.241 (10)	0.020 (5)	−0.005 (6)
O4	0.0625 (17)	0.0489 (15)	0.0459 (15)	−0.0062 (12)	0.0086 (12)	−0.0015 (12)
O5	0.070 (3)	0.052 (2)	0.078 (3)	0.000	−0.008 (2)	0.000

### Geometric parameters (Å, °)

**Table:**

Cu1—N6^i^	1.998 (2)	C3—C4	1.512 (5)
Cu1—N6^ii^	1.998 (2)	C3—H3A	0.9700
Cu1—N3^iii^	2.035 (3)	C3—H3B	0.9700
Cu1—N3	2.035 (3)	C4—C5	1.526 (5)
Cu1—O4	2.456 (3)	C4—H4A	0.9700
N1—C2	1.319 (4)	C4—H4B	0.9700
N1—N2	1.352 (4)	C5—H5A	0.9700
N1—C3	1.462 (4)	C5—H5B	0.9700
N2—C1	1.306 (4)	C6—H6	0.9300
N3—C2	1.324 (4)	C7—H7	0.9300
N3—C1	1.355 (4)	N7—O3'	1.164 (6)
N4—C7	1.318 (4)	N7—O1'	1.180 (5)
N4—N5	1.357 (4)	N7—O3	1.199 (11)
N4—C5	1.452 (4)	N7—O2'	1.232 (5)
N5—C6	1.304 (4)	N7—O1	1.253 (10)
N6—C7	1.319 (4)	N7—O2	1.261 (10)
N6—C6	1.350 (4)	O4—H4'	0.8499
N6—Cu1^iv^	1.998 (2)	O4—H4"	0.8500
C1—H1	0.9300	O5—H5	0.8500
C2—H2	0.9300		
			
N6^i^—Cu1—N6^ii^	180.0	C3—C4—C5	114.4 (3)
N6^i^—Cu1—N3^iii^	89.71 (11)	C3—C4—H4A	108.7
N6^ii^—Cu1—N3^iii^	90.29 (11)	C5—C4—H4A	108.7
N6^i^—Cu1—N3	90.29 (11)	C3—C4—H4B	108.7
N6^ii^—Cu1—N3	89.71 (10)	C5—C4—H4B	108.7
N3^iii^—Cu1—N3	180.0	H4A—C4—H4B	107.6
N6^i^—Cu1—O4	88.00 (10)	N4—C5—C4	113.1 (3)
N6^ii^—Cu1—O4	92.00 (10)	N4—C5—H5A	109.0
N3^iii^—Cu1—O4	92.03 (10)	C4—C5—H5A	109.0
N3—Cu1—O4	87.97 (10)	N4—C5—H5B	109.0
C2—N1—N2	110.5 (3)	C4—C5—H5B	109.0
C2—N1—C3	128.6 (3)	H5A—C5—H5B	107.8
N2—N1—C3	120.9 (3)	N5—C6—N6	114.1 (3)
C1—N2—N1	102.5 (3)	N5—C6—H6	122.9
C2—N3—C1	103.0 (3)	N6—C6—H6	122.9
C2—N3—Cu1	128.1 (2)	N4—C7—N6	109.6 (3)
C1—N3—Cu1	128.6 (2)	N4—C7—H7	125.2
C7—N4—N5	110.1 (3)	N6—C7—H7	125.2
C7—N4—C5	128.3 (3)	O3'—N7—O1'	124.8 (5)
N5—N4—C5	121.6 (3)	O3'—N7—O3	87.6 (11)
C6—N5—N4	102.7 (3)	O1'—N7—O3	125.8 (13)
C7—N6—C6	103.5 (3)	O3'—N7—O2'	117.6 (5)
C7—N6—Cu1^iv^	128.7 (2)	O1'—N7—O2'	117.6 (5)
C6—N6—Cu1^iv^	127.7 (2)	O3—N7—O2'	54.1 (11)
N2—C1—N3	114.4 (3)	O3'—N7—O1	148.4 (13)
N2—C1—H1	122.8	O1'—N7—O1	47.2 (12)
N3—C1—H1	122.8	O3—N7—O1	122.6 (9)
N1—C2—N3	109.6 (3)	O2'—N7—O1	79.3 (10)
N1—C2—H2	125.2	O3'—N7—O2	35.7 (10)
N3—C2—H2	125.2	O1'—N7—O2	93.3 (10)
N1—C3—C4	113.2 (3)	O3—N7—O2	122.0 (9)
N1—C3—H3A	108.9	O2'—N7—O2	144.5 (12)
C4—C3—H3A	108.9	O1—N7—O2	115.4 (8)
N1—C3—H3B	108.9	Cu1—O4—H4'	129.2
C4—C3—H3B	108.9	Cu1—O4—H4"	113.7
H3A—C3—H3B	107.8	H4'—O4—H4"	117.1
			
C2—N1—N2—C1	−0.1 (3)	C3—N1—C2—N3	−178.7 (3)
C3—N1—N2—C1	178.5 (3)	C1—N3—C2—N1	0.5 (3)
N6^i^—Cu1—N3—C2	71.7 (3)	Cu1—N3—C2—N1	−175.0 (2)
N6^ii^—Cu1—N3—C2	−108.3 (3)	C2—N1—C3—C4	102.6 (4)
N3^iii^—Cu1—N3—C2	142 (3)	N2—N1—C3—C4	−75.7 (4)
O4—Cu1—N3—C2	−16.3 (3)	N1—C3—C4—C5	−65.6 (4)
N6^i^—Cu1—N3—C1	−102.6 (3)	C7—N4—C5—C4	122.2 (4)
N6^ii^—Cu1—N3—C1	77.4 (3)	N5—N4—C5—C4	−58.5 (4)
N3^iii^—Cu1—N3—C1	−32 (4)	C3—C4—C5—N4	108.7 (3)
O4—Cu1—N3—C1	169.4 (3)	N4—N5—C6—N6	−0.2 (4)
C7—N4—N5—C6	0.3 (4)	C7—N6—C6—N5	0.0 (4)
C5—N4—N5—C6	−179.1 (3)	Cu1^iv^—N6—C6—N5	−178.7 (2)
N1—N2—C1—N3	0.4 (4)	N5—N4—C7—N6	−0.2 (4)
C2—N3—C1—N2	−0.5 (4)	C5—N4—C7—N6	179.1 (3)
Cu1—N3—C1—N2	174.9 (2)	C6—N6—C7—N4	0.1 (4)
N2—N1—C2—N3	−0.2 (4)	Cu1^iv^—N6—C7—N4	178.9 (2)

Symmetry codes: (i) *x*+1/2, *y*+1/2, *z*; (ii) −*x*+3/2, −*y*+1/2, −*z*; (iii) −*x*+2, −*y*+1, −*z*; (iv) *x*−1/2, *y*−1/2, *z*.
